# Heteroclinic networks for brain dynamics

**DOI:** 10.3389/fnetp.2023.1276401

**Published:** 2023-11-08

**Authors:** Hildegard Meyer-Ortmanns

**Affiliations:** ^1^ School of Science, Constructor University, Bremen, Germany; ^2^ Complexity Science Hub Vienna, Vienna, Austria

**Keywords:** cognitive processes, metastable states, chunking dynamics, binding problem, heteroclinic networks, generalized Lotka–Volterra equations, information processing, brain dynamics

## Abstract

Heteroclinic networks are a mathematical concept in dynamic systems theory that is suited to describe metastable states and switching events in brain dynamics. The framework is sensitive to external input and, at the same time, reproducible and robust against perturbations. Solutions of the corresponding differential equations are spatiotemporal patterns that are supposed to encode information both in space and time coordinates. We focus on the concept of winnerless competition as realized in generalized Lotka–Volterra equations and report on results for binding and chunking dynamics, synchronization on spatial grids, and entrainment to heteroclinic motion. We summarize proposals of how to design heteroclinic networks as desired in view of reproducing experimental observations from neuronal networks and discuss the subtle role of noise. The review is on a phenomenological level with possible applications to brain dynamics, while we refer to the literature for a rigorous mathematical treatment. We conclude with promising perspectives for future research.

## 1 Introduction

Reproducible sequential neural activity is experimentally found in sequential memory processes and fast recognition of stimuli by olfactory and auditory sensory systems. It is reproducible in the sense that similar stimuli lead to similar sequences. Examples are the insect olfactory system (Laurent et al., 2001; Galán et al., 2004), vocal tract of songbirds (Hahnloser et al., 2002), and mollusk sensory motor system (Varona et al., 2002; Levi et al., 2004). One of the first experimental hints for features of what is nowadays called winnerless competition was the complex intrinsic dynamics in the antennal lobe of insects that transform static sensory stimuli into spatiotemporal patterns of neural activity (Laurent et al., 2001; Galán et al., 2004). Winnerless competition is an overarching concept which, when applied to neurodynamics, means that, temporarily, certain neurons or subpopulations of neurons become dominant, while others are silent so that the winner of a competition changes with time. Many hints exist that typical states in the brain are metastable (Tognoli and Kelso, 2014), and the structure of these metastable states is reflected in functional neuroimage experiments. In particular, with respect to cognitive processes, common phenomenological features of brain dynamics are (i) that sensory information is coded both in space and time (!); (ii) cognitive modes do sensitively depend on the stimulus and the executed function; (iii) cognitive behavior is deterministic if the environment is the same and highly reproducible; and (iv) cognitive modes are robust against noise of diverse origin (Rabinovich et al., 2008a). Cognitive phenomena rely on transient dynamics such as working memory and decision making (Rabinovich et al., 2006a; Rabinovich et al., 2008b; Rabinovich et al., 2012), besides spatiotemporal sensory encoding (Rabinovich M. et al., 2008) and robust rhythmic generation (Rabinovich et al., 2006b).

Based on these observations, a mathematical framework should satisfy the following requirements: • in mathematical terms, the dynamics should be dissipative so that its orbits rapidly forget its initial state as soon as the stimulus is present; • variables should represent neural activities in brain modes such as firing rates; • the dynamical equations should have solutions that amount to metastable states; • the order of sequential switching between such states must be robust and based on mutual interaction between many individual cells; yet, the dynamics should be very sensible to external input; • the dynamics should be controlled by inhibition (in particular, for cognitive processes) and combined and balanced by excitatory interactions; and • it should be sensitive to prior neural or ongoing environmental input, so it should be possible to implement memory (Rabinovich et al., 2015a).

Naturally, the question arises as to which mathematical framework is suited to satisfy as many of these postulates as possible. Certainly, concepts from asymptotic dynamics, that is, long-time limits, large volumes, and (thermodynamic) equilibrium situations, are not suited, although in these limiting cases, the mathematics usually simplifies considerably. The simplest long-term asymptotic dynamics is a stable equilibrium (in the sense of a fixed point of dynamical systems theory rather than of thermodynamic equilibrium), or a stable limit cycle, or a chaotic attractor. These are typical invariant sets, in which the system remains forever unless parameter values change or other external perturbations occur, but activities of our mind are intrinsically transient and thoughts are elusive, even if they are well reproducible.

Nevertheless, in a first attempt, one may start with coding via a number of different fixed-point attractors. For example, what would such a coding mean for the olfactory system? Coding with attractors of Hopfield networks (Floréen and Orponen, 1989; Gopalsamy and He, 1994; Maass and Natschläger, 1997) would imply that each odor is represented by a specific behavior (attractor) of the neural network. The number of different attractors would determine the number of distinguishable stimuli that can be represented and recognized. Obviously, the system must be multistable, and for each odor, an attractor should be generated. Once the attractor landscape has formed in a Hopfield network, it is static after convergence. It is also robust or resistant to corruption of the input unless it is overloaded. However, the number *m* of stimuli allowed in a system of *N* neurons is very limited *m* < 0.14 *N* (Hertz et al., 1991) due to boundaries between basins of attraction which should not overlap; otherwise, the error rates in the retrieval of patterns of stimuli are large.

Other neurodynamic frameworks are coordination dynamics (Kelso, 1995; Rabinovich et al., 2001; Bressler, 2003; Tognoli and Kelso, 2014) and chaotic itinerancy (Kaneko and Tsuda, 2003; Tsuda, 2015), networks of Milnor attractors (Kaneko, 1998; Ashwin and Timme, 2005), cycling chaos with connections between chaotic saddles (Dellnitz et al., 1995; Ashwin and Rucklidge, 1998), and, last but not the least, HD with winnerless competition (Rabinovich and Varona, 2011; Ashwin et al., 2016a). Common to these approaches, neural computation is mediated by reproducible spatiotemporal patterns that amount to attempts of providing a mathematical solution. The specific realization of these accounts differs in what the states are in the mathematical description: fixed points in Hopfield models (Hopfield, 1982; Cohen and Grossberg, 1983), attractor ruins in chaotic itinerancy (Tsuda, 2015), and saddles, such as saddle equilibria, connected by heteroclinic orbits in HD, as further discussed in this review.

In contrast to Hopfield models, in heteroclinic networks (HNs), the repertoire of possibly stored patterns is considerably increased; since time is used as an additional coordinate for storage of information, the patterns are spatiotemporal and dynamically retrievable. For brain dynamics, heteroclinic networks are not a description on a neurophysiological level; neither are they restricted to a specific spatial or temporal scale of the brain or to a specific brain area nor do they account for physical aspects related to energy consumption or metabolism. What this framework of dynamical systems theory is suited for is a description of switching events where the considered system, such as a neuron population, spends some dwell time in a certain state and suddenly switches to another state. This is the case when brain dynamics proceeds via sequential segmentation of information that is manifest in sequences of electroencephalography (EEG) microstates (Michel and Koenig, 2018). In relation to motor processing in the brain, heterocyclic cycles are supposed to provide a possible explanation for various gaits in animal and human motion. In view of perception and learning and cognitive processes, HD captures the intrinsically transient nature of these processes, together with their precise reproducibility and the option of a low-dimensional encoding in spatiotemporal patterns.

This review is organized as follows. Section 2 provides some basic notions and definitions needed for understanding HD on a phenomenological level, while we refer to the literature for mathematically rigorous definitions and notions of stability. Section 3 focuses on a realization of HD via generalized Lotka–Volterra (GLV) equations; here, we concretize this abstract concept and provide examples from typical realizations. We discuss binding and chunking dynamics in phase space, dimensional reduction and synchronization of heteroclinic networks if assigned to a spatial grid, and entrainment to heteroclinic motion via pacemakers. Section 4 is devoted to the design of heteroclinic networks and its generalizations toward the inclusion of excitable networks in phase space. A possible goal of such constructions is to explain and reproduce features from experiments such as switching statistics, for which we provide some examples, in particular, from chaotic heteroclinic networks. The role of noise in relation to HD can be rather versatile and subtle, as discussed in Section 5, together with an application of external driving. We conclude in Section 6 with an outlook to promising perspectives for further work. For excellent extended reviews which include sections on HD, we refer to previous studies (Rabinovich and Varona, 2011; Ashwin et al., 2016a).

## 2 Basic definitions, notations, and stability issues of heteroclinic networks

**FIGURE 1:**
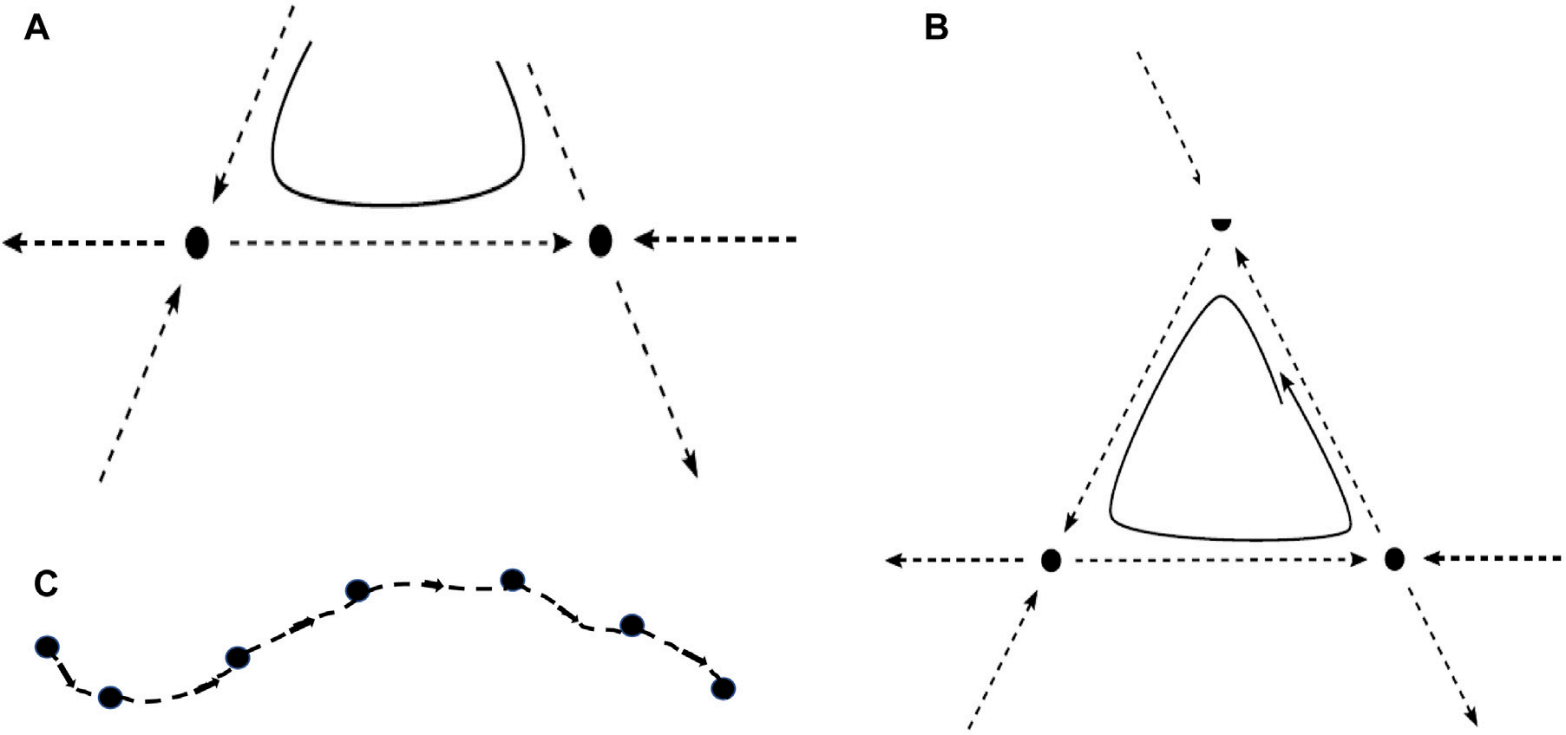
**(A)** Two saddle equilibria (full dots) with a heteroclinic connection (along the dashed line connecting them) and a possible trajectory (full line) in its vicinity. **(B)** Heteroclinic cycle between three saddle equilibria, composed of connections along the three dashed lines of the triangle, and a trajectory (full line) approaching the heteroclinic cycle. **(C)** Heteroclinic sequence, connecting a number of saddles. In all cases **(A–C)**, the unstable direction of one saddle becomes the stable direction of the subsequent one.

**FIGURE 2:**
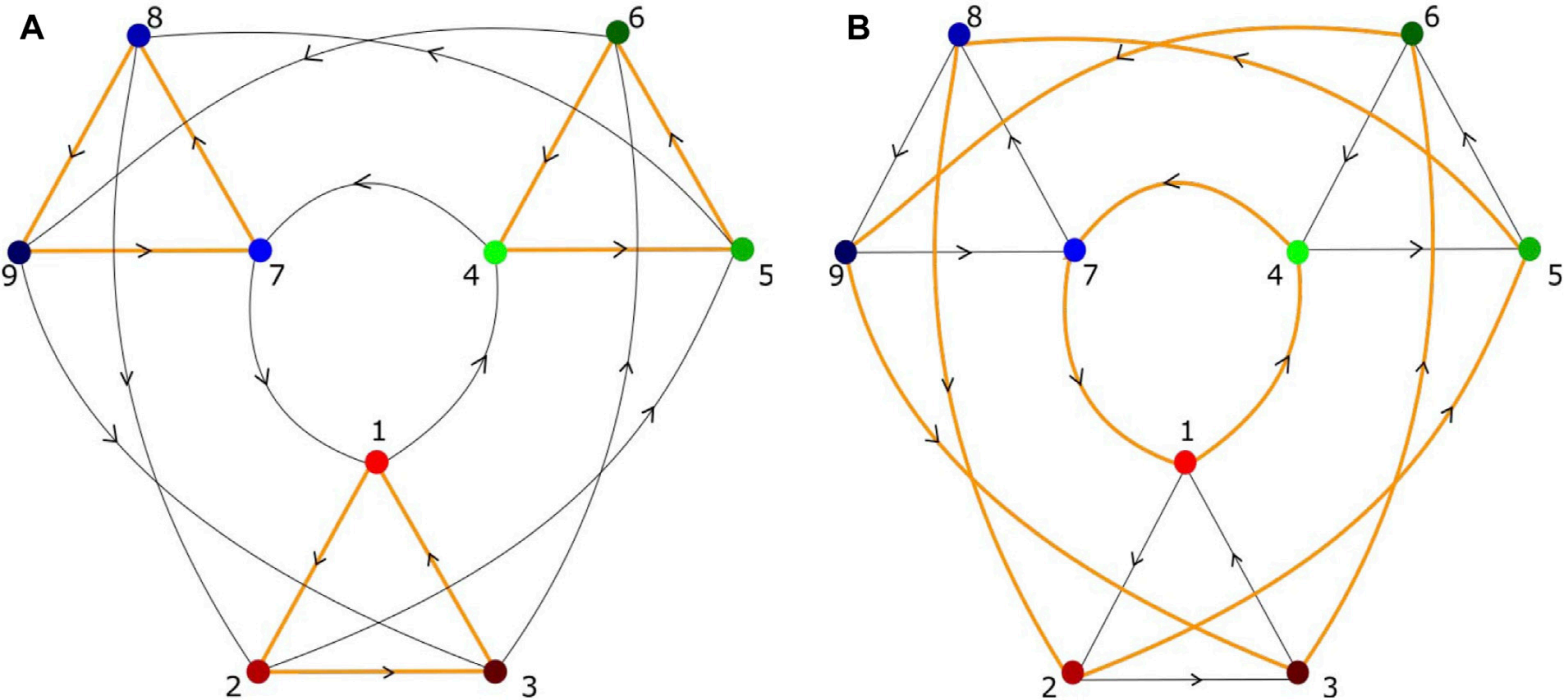
Heteroclinic network, composed of heteroclinic cycles, with nine saddles and possible heteroclinic connections between the vertices, but different heteroclinic network attractors **(A,B)**. Each saddle has two unstable directions, different from those shown in Figure 1. **(A)** Three small heteroclinic cycles (orange triangles), connected by heteroclinic connections of large heteroclinic cycles (black triangles). **(B)** Three large heteroclinic cycles (orange triangles) connected by small heteroclinic cycles (black triangles). **(A)** A possible sequence of visited saddles is (1,2,3) → (6,4,5) → (8,9,7), in **(B)** (1,4,7) → (8,2,5) → (6,9,3). Each saddle represents a metastable state in which a certain subpopulation of neurons is temporarily excited (Thakur and Meyer-Ortmanns, 2022).

When the unstable manifold of a saddle equilibrium intersects the stable manifold of another saddle, the intersection is called a heteroclinic connection (or orbit) (Figure 1A). A heteroclinic cycle (HC) is a closed loop in phase space consisting of a sequence of heteroclinic connections (Figure 1B). Figure 1C shows a heteroclinic sequence. An entire HN is a set of vertices, representing saddles, connected by edges, which are heteroclinic connections (Figure 2). It should be noticed that “saddles” are not restricted to saddle equilibria but refer to any invariant set, possessing non-trivial stable and unstable manifolds. When heteroclinic dynamics (HD) as HCs or HNs are assigned to a single site of a spatial grid, we term the corresponding ordinary differential equation (ODE) a heteroclinic unit (HU).

To become more precise, in the literature, different versions of definitions of HNs coexist. A compact one is as follows: consider an ODE $\dot{x}=f\left(x\right)$ with smooth *f*(*x*) that defines a flow on $x\in R^{d}$ . A closed invariant set $X\subset R^{d}$ is an HN for this flow if it is the union of a finite set of equilibria, such that the invariant set is chain transitive (Ashwin and Field, 1999; Ashwin and Postlethwaite, 2013). We refer to alternative definitions in Supplementary Appendix. The HN *X* is robust if (subject to specified constraints such as symmetries and smoothness) there is an open set of perturbations of *f* that have a nearby HN that is homeomorphic to the original. The HN is an attractor if it is an attractor in some well-defined sense, such as being asymptotically stable. It should be noticed that the notion of a network attractor as introduced by Ashwin et al. (2016b) refers to an invariant object in phase space that comprises not only local invariant sets but also the interconnections between them. An HN attractor is just a special case, the excitable network attractor (Section 4.1) another one. In particular, in the presence of additive noise, trajectories starting in the vicinity of a noisy heteroclinic attractor remain close to it for long periods of time and realize what is termed a heteroclinic channel by Rabinovich et al. (2012).

Concerning the asymptotic stability of HNs (rather than of individual HCs), Ashwin and Postlethwaite (2013) made the following conjecture: a sufficient (but not necessary) condition for asymptotic stability of the HN is that all the contracting eigenvalues are greater in absolute value than all the expanding eigenvalues, and all transverse and radial eigenvalues are negative. Contracting (expanding) eigenvalues mean a negative (positive) eigenvalue in the direction associated with an incoming (outgoing) heteroclinic connection to an equilibrium.

When HNs are attractors, invariant subspaces allow the option of robust connections between the saddles. The subspaces may be of different origins, in particular fixed-point subspaces induced by symmetry. In a simple realization, one has to deal with equilibria or periodic orbits as nodes with unstable manifolds such that there is a robust saddle-to-sink connection between the nodes within some invariant subspace. Such HNs can be attracting as precisely specified in previous studies (Melbourne et al., 1989; Krupa, 1997; Krupa and Melbourne, 2004). For the special case of an HC connecting saddle equilibria *ξ*_1_, …, *ξ*_*n*_, a sufficient condition for its stability is $-\lambda_{s}^{\left(i\right)}/\lambda_{u}^{\left(i\right)}>1$ for all *i*, where $\lambda_{s}^{\left(i\right)}$ $\left(\lambda_{u}^{\left(i\right)}\right)$ is an eigenvalue in the stable (unstable) direction of *Df*(*ξ*_*i*_), respectively. Further discussions on stability issues are provided by Guckenheimer and Holmes (1988), Field and Swift (1991), and Kirk and Silber (1994).

Robust heteroclinic attractors can be found in various systems, for example, in phase oscillator networks,

$$\frac{d\theta_{i}}{dt}=\omega +\epsilon \overset{N}{\underset{j=1}{\sum }}w_{ij}H\left(\theta_{i}-\theta_{j}\right),$$ (1)

where *H*(*θ*) = sin(*θ* − *α*) − *r* sin(2*θ*) and *ϵw*_*ij*_ is the weight of the connection from node *j* to node *i* and parameters *α* and *r*. Bifurcations occur as a function of *α* and *r*, and robust heteroclinic attractors are found for large *N* (Hansel et al., 1993). For small *N*, one can only find attractive robust heteroclinic attractors for *N* ≥ 4 (Ashwin et al., 2008). Furthermore, Hodgkin–Huxley-type limit-cycle oscillators with delayed synaptic coupling show similar heteroclinic attractors (Ashwin et al., 2011). Another set of phase oscillator dynamics is considered by Ashwin et al. (2007), where various cluster states form a large HN that is an attractor and can serve to encode a variety of inputs such as heterogeneities in the natural frequencies (Orosz et al., 2009). Furthermore, locally coupled phase oscillators can lead to robust heteroclinic attractors if enough invariant subspaces exist for robust connections (Karabacak, Ashwin, 2010). In the following sections, we focus on HNs which realize the concept of winnerless competition in GLV equations.

## 3 Winnerless competition in GLV equations

When heteroclinic connections should realize a concept of winnerless competition, usually, GLV equations are considered. In the context of neuroscience, they describe the firing rate *A*_*i*_(*t*) of a neuron or a group of neurons. A number of different reviews exist on different versions of GLV equations and their accordingly varying interpretations in relation to brain dynamics, referring to cognition, emotion, attention, decision making, consciousness, creativity, and other modalities (Rabinovich et al., 2006a; Rabinovich and Muezzinoglu, 2010; Rabinovich and Varona, 2011; Rabinovich et al., 2012; Rabinovich et al., 2015b; Rabinovich et al., 2020). Here, we start with a generic set of these equations and indicate its general structure and meaning of different terms. It reads

$$\tau_{i,m}\frac{d}{dt}A_{i}^{m}\left(t\right)=A_{i}^{m}\left(t\right)\left[\sigma_{i}\left(S,A^{n},R\right)-\overset{N}{\underset{j=1}{\sum }}\rho_{ij}\left(A^{n},R\right)A_{j}^{m}\left(t\right)\right]+A_{i}^{m}\left(t\right)\eta \left(t\right),\;i=1,\ldots ,N\;m=1,\ldots ,L$$ (2)

for the i-th neural activity $A_{i}^{m}$ of modality *m*. If a single modality, such as cognition, is considered with only one relevant time scale, the time constant *τ*_*i*,*m*_ would be absorbed on the right-hand side; here, it is spelled out to indicate the applicability to different time scales and the possibility of coexisting time scales, in particular of other modalities. In Eq. (2) we explicitly indicated other possible modalities $A^{n},n\neq m\in \left\{1,\ldots ,L\right\}$ . The excitatory first term depends on $\sigma_{i}\left(S,A^{n},R\right)$ , which is, in general, a function of the sensory input S , possible resources R , and neural activities of other modalities $A^{n}$ . If $A^{m}$ represents cognition, $A^{n}$ may represent emotion, and for $A^{n}$ , here, a GLV equation of the same type is postulated as in Eq. 2. The second inhibitory term in Eq. (2) represents the competition for resources and contains couplings $\rho_{ij}\left(A^{n},R\right)$ , which may be functions of resources and the nodes of other modalities. In particular, the inhibitory term can be split into inhibitory couplings between modes of the same modality or different modalities. An example for such a splitting is given in Eq. 4. In general, a multiplicative noise term is added, specific to the dynamics on the respective time scale *τ*_*i*,*m*_ of modality *m* with *η*(*t*) often chosen as Gaussian white noise. When GLV equations for different modalities are coupled, they usually differ in the HD; they need not even admit heteroclinic sequences individually. Resources R or external input S are assumed to obey their own dynamics, again of the GLV type or different ones such as gradient dynamics as in the study by Rabinovich et al. (2006c).

In the following sections, we discuss a few simple special cases without explicit modeling of the dependence of the excitatory and inhibitory couplings on external input, resources, or other modalities. Equation 2 then reduces to

$$\frac{dA_{i}}{dt}=A_{i}\left(\sigma_{i}-\overset{N}{\underset{j=1}{\sum }}\rho_{ij}A_{j}\right)$$ (3)

in a simple but important special case. Already, since the work of May and Leonard (1975), it is known that Eq. 3 can have robust heteroclinic attractors for *N* ≥ 3 and for an open set of parameter choices *σ*_*i*_ and *ρ*_*ij*_. The variety of equilibria range from coexistence equilibria where several *A*_*i*_ are different from zero to temporary winner-takes-all (*A*_*i*_ > 0 and *A*_*j*_ = 0 for all *j* ≠ *i*) to periodic or chaotic dynamics. The winnerless competition amounts to sequences of saddles joined by robust connections. They are robust because of the lack of firing of one neuron or a group of neurons, since *A*_*i*_ = 0 is preserved by the dynamics (Hahnloser et al., 2002). A simple example for winnerless competition for *N* = 3 corresponds to a rock–paper–scissors game with cyclic inhibition of the neurons in one direction of the ring and cyclic excitation in the opposite direction such that *dA*_*i*_/*dt* = *A*_*i*_(1 − *A*_*i*_ − *αA*_*i*+1_ − *βA*_*i*+2_) with *i* = 1, 2, 3 mod 3, and *α* + *β* > 2, 0 < *α* < 1 (May and Leonard, 1975). The local behavior near this type of heteroclinic attractors was termed stable heteroclinic channels (Rabinovich et al., 2012). As further special cases of Eq. 2 we discuss binding and chunking dynamics in the following subsections because of their physical meaning with respect to brain dynamics.

### 3.1 Binding dynamics

A widely discussed question in neuroscience is the binding problem, formulated and reviewed by Von der Malsburg (1999). It refers to the need of a coherent representation of an object provided by associating all its features such as shape, color, sound, smell, taste, location, and speed. Only the binding of all these features allows a unified perception of the respective object. Von Der Malsburg and Schneider (1986) suggested that temporal synchrony of neural activities may provide a solution of the binding problem. Neurons can be temporary members of different cell assemblies at different instants of time. In relation to HD, a possible realization of binding may be two HCs, representing two modalities, such as visual and auditory ones, “bound” by heteroclinic connections (between these cycles) and heteroclinic sequences within these cycles, which become synchronized. This may realize the coordinated perception of visual and auditory inputs as belonging to the same object.

A concrete mathematical implementation was discussed by Rabinovich et al. (2010) and Afraimovich et al. (2015) and given as

$$\frac{dA_{i}^{l}}{dt}=A_{i}^{l}\left(\sigma_{i}^{l}-\overset{N}{\underset{j=1}{\sum }}\rho_{ij}^{l}A_{j}^{l}-\overset{L}{\underset{m=1}{\sum }}\overset{N}{\underset{j=1}{\sum }}\xi_{ij}^{lm}A_{j}^{m}\right),$$ (4)

where *i* = 1, …, *N*, *l* = 1, …, *L*, and $A_{i}^{l}$ is the activity level of the i-th mode in the l-th informational modality (visual, odor, auditory, etc.). The total number of modes from different brain areas is then *N*⋅*L*. The coupling parameters $\rho_{ij}^{l}$ describe the inhibitory connections between modes *i* and *j* in the same modality *l*, while $\xi_{ij}^{lm}$ refer to connections between mode *i* of modality *l* and mode *j* of modality *m*. Furthermore, $\sigma_{i}^{l}$ denotes the strength of the stimulation of the mode *i* in the l-th modality. In this formulation, the parameters $\xi_{ij}^{lm}$ control the options for possible binding. The authors prove the existence of heteroclinic channels in the neighborhood of the considered HNs. For *L* = 3 modalities (of what is termed sequential memory), it was numerically illustrated by Afraimovich et al. (2015) that the dynamics is sensitive to external input, robust, and reproducible, and in particular, that HCs exist which are joined (“bound”) by heteroclinic connections.

It should be noted that at least two-dimensional unstable manifolds are required at the saddles to allow the trajectory either to (preferably) remain in the same modality or (less preferably, but possibly) to escape along a heteroclinic connection to another modality. Higher-dimensional unstable manifolds are needed if a trajectory should have possible exits to a number of different modalities. The conjecture is that winnerless competition (if realized in this framework of binding dynamics) plays a basic role in different kinds of sequential memory (episodic or working memory), i.e., memory that has to deal with a sequential order of thoughts or events which need to be “bound together” to achieve higher levels of cognition.

### 3.2 Hierarchical HNs, chunking dynamics, and magic numbers

As mentioned previously, the idea is that brain activity is organized in spatiotemporal patterns through transient metastable states (Kelso, 1995; Friston, 1997), in particular the processing of sequential cognitive activity. Particularly during cognition, it is evident that the brain does select a subset of relevant metastable states, suppressing the irrelevant ones in view of a given task, while it relies on hierarchical organization of the global brain networks. In view of reproducing effectively low-dimensional brain dynamics, the external and internal stimuli leading to a cognitive task should be efficiently encoded, and only a moderate number of brain excitation modes should become excited upon the performance.

Here, the concept of hierarchical HNs is a suitable framework as it allows us to describe chunking dynamics. Chunking is a widely observed phenomenon in processes such as perception, learning, and cognition. It refers to splitting long information sequences into shorter parts, so-called chunks, for better storing and processing the information. It is similar to the structure of language, composed of novels, sections, sentences, and words, the rhythm in poems, or, more banal, from memorizing credit card numbers as four chunks of four numbers each. The concept of chunks goes back to the work of Miller (1956). Chunking consists of the segmentation of long strings into shorter segments as well as the concatenation of these segments. Obviously, such a procedure can be iterated a number of times and is intrinsically hierarchical. This is a desired feature in view of the experimental facts that activities of functional networks in the brain are hierarchically organized if one has to deal with perception, cognition, behavioral sequential activity or motor control (Grossman, 1980; Rosenbaum et al., 1983; Musso et al., 2003; Tettamanti and Weniger, 2006), or specific movements (Bahlmann et al., 2008; Bahlmann et al., 2009; Kotz et al., 2010). If a chunk or superchunk represents an entire block or a set of blocks on lower organization levels, this goes along with a strong reduction in the degrees of freedom which may be the relevant ones for the specific task.

A hierarchical HN is composed of hierarchically connected HCs or heteroclinic sequences, if we extend the definition of an HN to include sequences as a special case. A formal expression, as considered by Afraimovich et al. (2014), is sets of GLV equations, one for each hierarchy level, that is, in the case of three levels, the level of elementary information items, the level of chunks, and the level of superchunks. The hierarchy in time scales is explicitly implemented via parameters scaling the time in the corresponding equations. The variables interact within and between the different hierarchy levels. Important for the structural stability are the asymmetric inhibitory connections. A concrete example of chunking dynamics was given by Afraimovich et al. (2014) with three hierarchy levels. In a time series, the oscillating envelopes of elementary items are chunks, and the envelopes of chunks are superchunks, with each chunk here composed of six elementary items and each of the three superchunks composed of six chunks in the study by Afraimovich et al. (2014). This amounts to a reduction from 108 items on the elementary level to three degrees of freedom on the superchunk level. On the chunk level, fast oscillations are modulated by slow oscillations, and on the superchunk level, slow oscillations are modulated by super-slow oscillations, features as they are found in brain dynamics.

**FIGURE 3:**
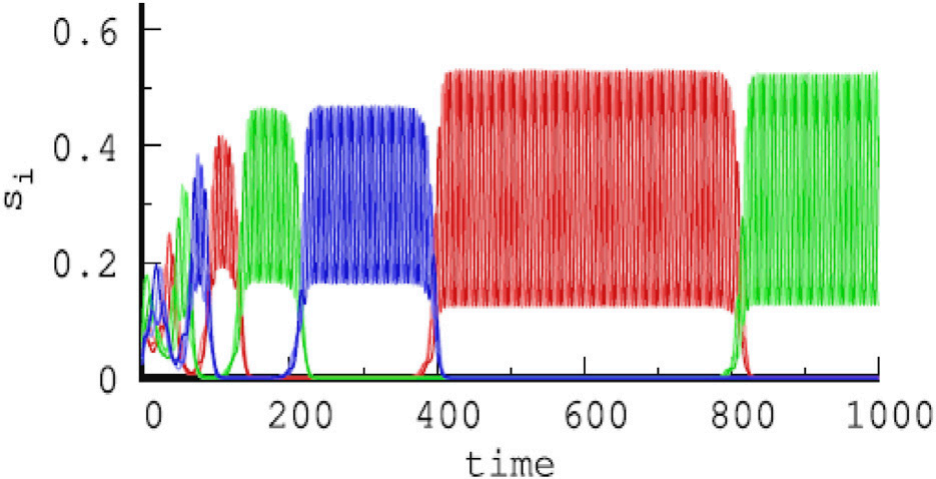
Temporal evolution of three different chunks (red, green, and blue) of increasing duration with time in a hierarchical HN, each chunk composed of fast oscillations between three shades of red, green, and blue, respectively, and corresponding to nine types of firing activities of different subpopulations, here, *s*_*i*_ ≡ *A*_*i*_ (Voit and Meyer-Ortmanns, 2020), where *s*_*i*_ was chosen in reminiscence of species in an ecological context.

**FIGURE 4:**
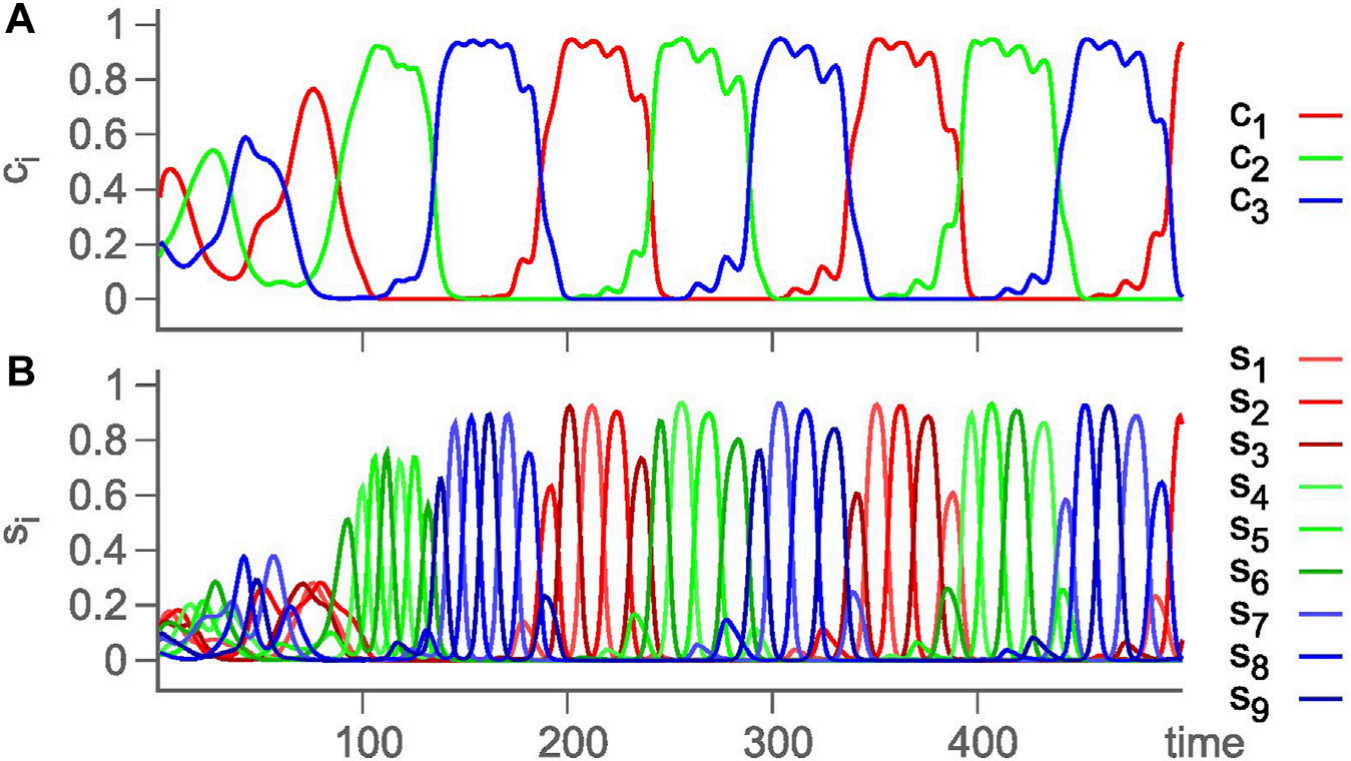
Again, chunking dynamics of a hierarchical HN with two levels of hierarchy, but in the presence of noise to prevent slowing down of the oscillations. **(A)** Cluster activity *c*_*i*_ = *∑*_1≤*j*≤3_ *s*_3(*i*−1)+*j*_, reflecting the chunks, and **(B)** time evolution of the underlying firing activity levels *s*_*i*_ ≡ *A*_*i*_ (Voit and Meyer-Ortmanns, 2019).

An alternative approach of modeling hierarchical HNs was pursued in previous studies (Voit and Meyer-Ortmanns, 2018; Voit and Meyer-Ortmanns, 2019a; Voit and Meyer-Ortmanns, 2019b; Voit and Meyer-Ortmanns, 2019; Voit and Meyer-Ortmanns, 2020), where the hierarchy in attractor space and in time scales was exclusively implemented in the rate matrix *ρ*_*ij*_ in a single GLV equation. The choice of rates or competition strengths was assumed to be selected by external or internal signals. The choice was such that the absolute values of eigenvalues of contracting directions are larger than those for the expanding directions, and eigenvalues in the radial and transverse directions are negative. According to the conjecture of Ashwin and Postlethwaite (2013), this choice realizes sufficient conditions for asymptotic stability of the embedding HN (for details, see the work of Ashwin and Postlethwaite (2013) and Voit and Meyer-Ortmanns (2018)). This way, an HC of three HCs, each between three saddle equilibria, is constructed, with a long time scale corresponding to a revolution between the three HCs and a short time scale for a revolution between the three saddle equilibria, that is, chunking dynamics with two hierarchy levels. Figure 3 is from such a simulated chunking dynamics with three types of chunks (red, green, and blue) (Voit and Meyer-Ortmanns, 2020), similar to Figure 4 in the presence of noise to avoid slowing down upon approaching the HCs (Voit and Meyer-Ortmanns, 2019).

Bick and Rabinovich (2009) attempted to explain the dynamical origin of the effective storage capacity of the working memory on the basis of GLV equations . They derive conditions for allowing a stable heteroclinic channel, given a fixed stimulus and inhibitory couplings, randomly picked within certain bounds. The longer the sequence, which should be kept in the working memory, the more the need of excitation support, since longer sequences lead to the activation of more inhibitory connections. The ratio of randomly selected inhibitory connections relative to the lateral connections, which are responsible for the temporal order along the heteroclinic sequence, is shown to be lower bounded by a function that increases with the length of the sequence in terms of the number of saddles. On the other hand, the relative connection strengths are biologically also upper bounded. To satisfy the inequalities, this upper bound imposes a constraint on the maximal number of saddles that leads to a stable heteroclinic channel. The order of magnitude derived within this chunking dynamics model is compatible with Miller’s magic number 7 ± 2 (Miller, 1956), and, more importantly, the work illustrates a possible dynamical origin (in terms of stability) of the limited storage capacity in the working memory.

So far, we used the notion of “chunk” in the usual vague way as a set of items (oscillations) that are treated as a single unit on the chunking level. A quantitatively more precise conception was derived by Mathy and Feldman (2012) from the notion of Kolmogorov complexity and compressibility, in which a chunk is a unit in a maximally compressed code. In agreement with experiments, the authors conclude that the true limit of the short-term memory capacity is approximately three or four distinct chunks after compression, which is said to be equivalent to approximately seven uncompressed items of typical compressibility. This is consistent with both Miller’s magic number 7 ± 2 and, as more recently stated, 4 ± 1 chunks for the capacity of short-term memory.

### 3.3 Heteroclinic dynamics on spatial grids

HNs are networks in phase space. Already, the dynamics of small HNs may be rather rich with intricate bifurcation diagrams (Voit et al., 2020). Thus, one may wonder why to add spatial dimensions and assign HNs to a spatial grid. In general, the interest is in spatial patterns or in collective dynamics if many HNs of the same type are coupled. Spatial coupling is obviously relevant not only in ecological applications of population dynamics but also in neuronal dynamics when HCs are coupled from different areas of the brain for binding their provided information. Moreover, HNs on a spatial grid seem to realize a postulate of Grossberg (2000), that is, the “complementary brain” in contrast to a brain that is organized into modules. With examples from perception, learning, action, and cognition, Grossberg (2000) argued as to why the organizational structure should be described in terms of parallel processing streams with complementary properties, hierarchical interactions within each stream, and parallel interactions between the streams. In the framework of HNs assigned to a spatial grid, parallel processing may go on between different HNs, assigned to different spatial locations, while hierarchically organized streams are realized as hierarchical HNs in phase space.

Previous work on HNs on one- or two-dimensional spatial grids dealt with rock–paper–scissors games (Li et al., 2012; Postlethwaite and Rucklidge, 2017; Postlethwaite and Rucklidge, 2019) for small grids, spatiotemporal chaos in large one-dimensional grids with Lotka–Volterra equations (Orihashi and Aizawa, 2011), or the quasi-periodic route to chaos for such a set (Sprott et al., 2005). Voit et al. (2020) considered small sets of coupled HCs, which display a variety of dynamic behaviors such as limit cycles, slowing-down states, quasi-periodic motion, transient chaos, and chaos.

In view of brain dynamics, we focus on synchronization properties of spatially coupled hierarchical HNs. A simple way of introducing spatial interaction between such HNs is via diffusion,^1^ leading to the GLV equation of the form (Voit and Meyer-Ortmanns, 2020)

$$\partial_{t}A_{i}=\delta \nabla_{x}^{2}A_{i}+\sigma A_{i}-\gamma A_{i}^{2}-\underset{j\neq i}{\sum }\rho_{ij}A_{i}A_{j}+\eta |\xi_{i}\left(t\right)|,$$ (5)

where the competition rate matrix *ρ*_*ij*_ is chosen as in the study by Voit and Meyer-Ortmanns (2018) to induce a hierarchy in time scales, *δ* is the diffusion rate, $\nabla_{x}^{2}$ is the two-dimensional grid Laplacian on an *L* × *L*-grid, *σ* is chosen as 1 to set the time scale, *γ* denotes the death rate, *η* is the noise amplitude, and *ξ*_*i*_(*t*) is the Gaussian white noise. Coupling via diffusion amounts to an attractive interaction. Thus, it is not surprising that the HN can synchronize on a spatial grid to the dynamics of a single hierarchical HN. For weak diffusion, the hierarchy in time scales translates to a hierarchy in the generated spatial scales, visible in nested spirals whose arms are chasing each other if the time evolution is visualized in a movie. Given a grid size, for sufficiently strong diffusion, the synchronization proceeds such that, first, the large HCs at different sites choose the same small HC out of the three available ones in our example; next, the same saddle equilibrium out of the three saddles of the small HC. The entire grid then flips in synchrony between the nine different neuron populations, ending up with a uniform saddle choice on the entire grid (Voit and Meyer-Ortmanns, 2019). Such a strong dimensional reduction is of interest in view of brain dynamics as cognition makes heavy use of dimensionally reduced representations.

Possible effects of diffusive coupling may go far beyond simple synchronization of the entire grid. When its effect is studied for cyclic competition between four species in one dimension and the system is described in a steady-state traveling frame of reference, a bifurcation analysis, in particular as a function of the speed of the wave, becomes possible and reveals additional HNs with additional heteroclinic orbits due to the diffusive coupling (Dijkema and Postlethwaite, 2022). The traveling wave solutions amount to periodic orbits in ODEs in this new reference frame. Within this approach, it seems possible to reproduce the formation of alliances between players in cyclic competition games (Durney et al., 2011; Roman et al., 2012; Dobramysl et al., 2018; Dijkema and Postlethwaite, 2022) if the variables *A*_*i*_ represent concentrations of ecological or social species rather than neural activities. It seems open whether the formation of alliances in ecological or social systems has a counterpart of interpretation in cognitive processes. The dynamical framework allows a variety of such “games”.

### 3.4 Entrainment to heteroclinic motion

Diffusion amounts to a special type of attractive coupling, certainly not the only option for choosing couplings. Neither is a homogeneous choice of parameters *σ*, *γ*, *ρ*_*ij*_ and *η* all over the grid a realistic feature. For brain dynamics, the interest is, in general, in partial synchronization of subpopulations of neurons and under heterogeneous parameter conditions. In view of the latter aspect, the question arises as to whether HUs in a resting state may be entrained by other units, which are in the mode of heteroclinic cycling. As discussed by Thakur and Meyer-Ortmanns (2022), this is possible if we consider a set of HUs which individually perform (hierarchical) heteroclinic oscillations (the pacemakers), and these units are directionally coupled to units in a resting state (the driven units). Unless the driven units become entrained, individually, they would approach a coexistence equilibrium, termed the resting state. The coupling may be unidirectional or asymmetric bidirectional. The entrainment range turns out to depend on the type of coupling, the spatial location, individual bifurcation parameters of the pacemaker and the driven units.

**FIGURE 5:**
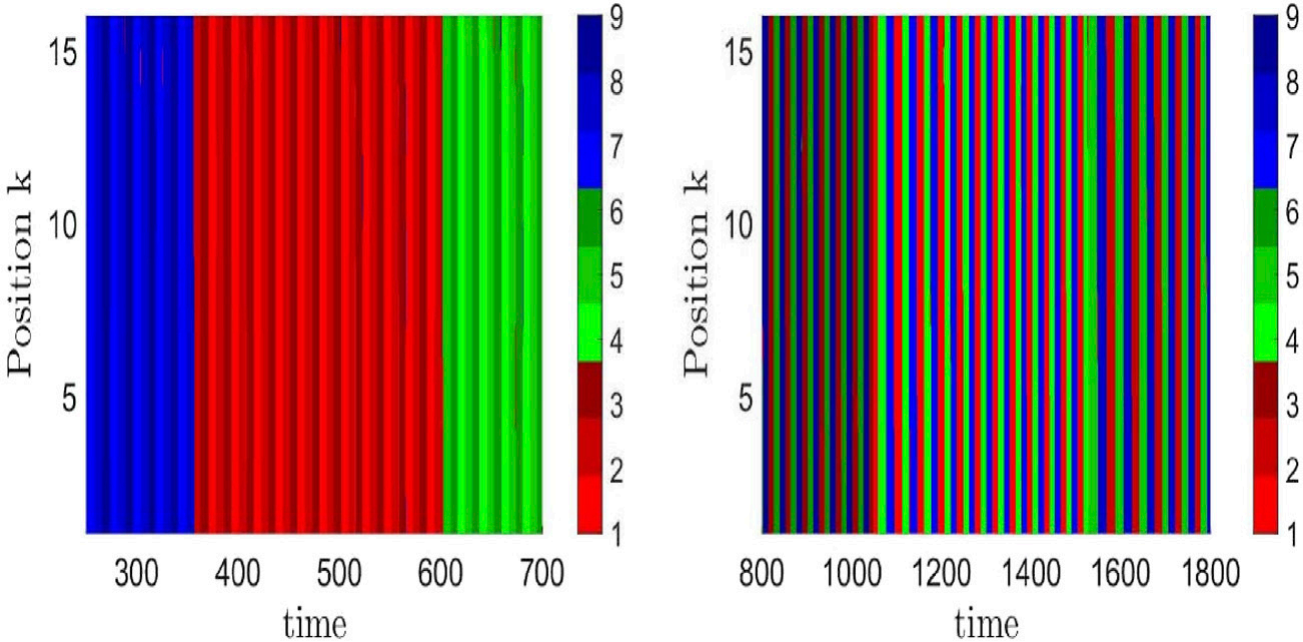
Time evolution of nine types of neural activity levels (three shades of red, blue, and green) in a chain of coupled hierarchical heteroclinic units: one pacemaker at site 1 of the chain and 15 driven units at the remaining 15 positions, completely synchronized (same color along a vertical stripe) to the motion of the pacemaker. The difference in the resulting patterns as a function of time in the right and left panels reflects different heteroclinic network attractors, here, corresponding to those of Figures 2A, B, respectively. This means that different sequences of the nine excitation patterns, supposed to encode information, are transferred over the grid as a result of entrainment (Thakur and Meyer-Ortmanns, 2022).

In view of entrainment, let us recall that information is encoded, in particular, in temporal patterns. The patterns are composed of a sequence of transiently excited neural populations, where the specificity of this information depends on the selected path of approached saddles in the HN of the pacemaker. If this pacemaker entrains units in a resting state to synchronize with its heteroclinic oscillations, this means that the temporal information of the pacemaker is processed over the spatial grid. An example of how the temporal information pattern differs as a function of the chosen HN attractor is shown in Figure 5 for two network attractors, characterized by the temporal sequence of visited saddles: left panel (1,2,3) → (6,4,5) → (8,9,7), right panel (1,4,7) → (8,2,5) → (6,9,3), where the integers label the saddles. These paths are schematically shown in Figure 2. The uniformly colored vertical stripes reflect the synchronization across the chain. If instead of a chain, the pacemakers are arranged in a local disc in the center of a two-dimensional grid, the processed information is visible as target waves emitted from the disc, where the information decays with the distance to the pacemakers (Thakur and Meyer-Ortmanns, 2022). Notably, the individual parameters of the resting units were chosen heterogeneously. It seems worthwhile to further explore how to control the synchronization patterns via the spatial coupling between pacemakers and driven units.

So far, we discussed HNs for realizing the concept of winnerless competition exclusively by means of GLV equations. Alternative to GLV equations, winnerless competition is found in coupled cell networks (Aguiar et al., 2011), or in delayed pulse-coupled oscillators (Kori, 2003; Neves and Timme, 2012), or designed by construction as demonstrated by Ashwin and Postlethwaite (2013), or along with excitable networks (Ashwin et al., 2016b). Design of HNs is discussed in the following section.

## 4 Designing heteroclinic networks as desired

Given the experimental data, it may not be possible to directly identify the relevant degrees of freedom or the metastable states. In such a case, data-driven modeling may first be applied, such as principal component analysis (Kato et al., 2015; Kutz et al., 2016; Brunton and Kutz, 2019), hidden Markov models (Westhead et al., 2017), or, more recently, a Koopman approximation (Klus et al., 2020). However, a theoretical approach in terms of designed HNs is promising if the dimension of the considered system can be reduced, when it has a finite number of dominant metastable states and a focus on a small set of degrees of freedom is possible. Coupled HNs often come in disguise in the sense that they manifest themselves as limit cycles, slowing-down states, quasi-periodic motion, transient chaos, and chaos, although the building blocks of the dynamics are only a few coupled HCs, such as two or three per site, or small networks of two units, each consisting of two coupled HCs. The dynamics is very flexible and versatile (Voit et al., 2020). Therefore, an attempt to reproduce experimental data in terms of the designed HD is promising: forming an HN by realizing heteroclinic connections between saddles as the main building blocks of the dynamics comprises a rich variety of dynamical features.

In general, given a directed graph, in our context with nodes corresponding to metastable states and with edges representing possible transitions between these states, it is far from unique to find a dynamical representation in terms of ODEs, which means that the vector fields are not unique which can support the existence of an HN corresponding to this graph. Therefore, on the mathematical side, a number of constructions of HNs have been proposed related to homogeneous or heterogeneous cell systems (Field, 2015), patterns of desynchronization and resynchronization (Field, 2016), discrete and continuous dynamics (Weinberger et al., 2018), HNs with specific properties such as robustness, completeness, and equability (Ashwin et al., 2020), or low-dimensional realizations of systems in spite of many equilibria and possible connections (Castro and Lohse, 2023). One may look for applications of such constructions to brain dynamics and search for mathematical properties that have a counterpart in properties from brain dynamics.

In the following sections, we present two approaches in more detail, which are of interest in relation to brain dynamics; the first one amounts to designing (noisy) heteroclinic networks from graphs (with extensions to excitable networks) (Ashwin and Postlethwaite, 2013; Ashwin and Postlethwaite, 2016; Ashwin et al., 2016b; Ashwin and Postlethwaite, 2018; Ceni et al., 2020; Creaser et al., 2021), and the second one amounts to constructing chaotic heteroclinic networks (Morrison and Young, 2022). In the latter, chaotic features of a deterministic description reproduce randomly looking decisions in the data.

### 4.1 Designing heteroclinic networks from graphs

Hierarchical HNs, as considered by Voit and Meyer-Ortmanns (2018), were already designed to reproduce a hierarchy in time scales, where slow oscillations modulate fast oscillations, and when assigned to a spatial grid, a hierarchy in spatial scales is ensued. The construction via an appropriate choice of rates was based on the work by Ashwin and Postlethwaite (2013) and their conjecture that HNs are asymptotically stable for an appropriate choice of eigenvalues, as indicated in Section 2. Let us go into more detail of the work by Ashwin and Postlethwaite (2013) and assume that motivated by experimental observations, we want to consider a particular directed graph whose nodes correspond to metastable states and want to realize this graph as an attracting robust HN between these states in phase space. Ashwin and Postlethwaite (2013) presented two methods of realizing such a graph as a robust HN for flows, generated by ODEs, where the vertices of the graph represent the saddle equilibria and the edges represent the heteroclinic connections between the vertices. Here, we sketch only one of the two methods, the so-called simplex realization that assumes graphs free of one cycle and two cycles. The graph is then realized as an invariant set within an attractor, and the flow of the vector field on $x\in R^{n_{V}}$ obeys the ODE

$$\frac{dx_{j}}{dt}=x_{j}\left(1-|x{|}^{2}+\overset{n}{\underset{i=1}{\sum }}a_{ij}x_{i}^{2}\right)+\zeta w_{j}\left(t\right),$$ (6)

where $x_{j}\in R$ , *j* = 1, …, *n*_*v*_, *n*_*v*_ is the number of vertices, $|x{|}^{2}=\sum_{j}x_{j}^{2}$ , and *w*_*j*_ represents i.i.d. white noise with amplitude 0 ≤ *ζ*. The claim then is that for *ζ* = 0, the rates *a*_*ij*_ can be chosen so that the graph has an HN *X*. This realization is robust to perturbations that respect the symmetry given by reflection in the coordinate planes. It should be noted that the proposition of Ashwin and Postlethwaite (2013) provides an open set of functions that lead to embedding in a heteroclinic attractor, not a set of unique parameters of *f* if *f* denotes the right-hand side of Eq. 6 apart from the noise term. However, the statistics of residence times in the vicinity of saddles and the transition probabilities at decision points are very sensitive to the actual choice of parameter values.

In extension of the work by Ashwin and Postlethwaite (2013), Ashwin and Postlethwaite (2016b) considered two types of cell networks, given by

$$\begin{array}{ccc} dp_{j} & = & \left[f\left(p_{j},y_{k}\right)\right]dt+\eta_{p}dw_{j},\\ dy_{k} & = & \left[g\left(p_{j},y_{k}\right)\right]dt+\eta_{y_{k}}dw_{k}^{\prime }, \end{array}$$ (7)

for *j* = 1, …, *M* and *k* = 1, …, *Q*, where *M* represents the number of states and *Q* is the number of possible transitions between states. Each cell type has its own dynamics, specified with concrete expressions for *f* and *g* by Ashwin and Postlethwaite (2016b). One type (p-cells) consists of a network of mutually inhibiting cells with multiple attractors, while the second one (y-cells) is made up of cells that selectively strongly excite certain states of p-cells while inhibiting other states of p-cells and simultaneously inhibiting each other; y-cells become active only when there is a transition between vertices. The quantities $w_{j}$ and $w_{k}^{\prime }$ are independent identically distributed noise processes with noise amplitudes *η*_*p*_ and $\eta_{y_{k}}$ . All this is in reminiscence to neuronal networks.

This way, the work provides an explicit construction of how any desired finite graph (without simple self-loops) can be realized as a network attractor between states in phase space, where the network is either heteroclinic or excitable in the following sense. In the excitable case, a perturbation exceeding a finite threshold (but possibly a very small one) is needed for a transition between stable fixed points, which are sensitive to perturbations in directions corresponding to the edges of the graph.^2^ In the heteroclinic case, transitions occur spontaneously without noise or external input, and the vertices are saddle equilibria with heteroclinic connections as edges. Remarkably, it is possible to tune the edges of the network from representing excitable connections to heteroclinic ones by a single parameter. While HN attractors are only stable as long as special structures in phase space, such as symmetries or invariant subspaces, protect them, network attractors joining stable fixed points do not encounter these restrictions.

Notable is, here, a possible application to brain dynamics. The simple switch of connections between heteroclinic and excitable behavior (due to the variation of a single parameter) may imitate a change in the concentration of neuromodulators such as dopamine, serotonin, or GABA (gamma-aminobutyric acid). Reducing the excitability of states in the model may represent the action of a neuromodulator that depresses excitability (Ashwin and Postlethwaite, 2016b).

The construction of Eq. 7 turns out to be very sensitive to noise in terms of which part of phase space is visited and in which order. Under the action of noise of strength *η*, the dwell time *T* near equilibria depends on whether we have to deal with the heteroclinic (*T* ∝ − ln *η*) or the excitable realization (given by Kramer’s law) (Ashwin and Postlethwaite, 2016). Application of anisotropic noise suggests that the construction allows the design of noisy attractors with arbitrary Markov transition rates and dwell times.

When it comes to using noisy attractor models for reproducing measured transitions between EEG microstates collected from healthy subjects at rest, the model of Eq. 7 can reproduce the transition probabilities between microstates but not the heavy-tailed dwell time distributions. For their reproduction, further extensions of the noisy network attractor model are needed. One option is with an additional hidden node at each state, while the second option is with an additional layer that controls the switching frequency in the original network. With these extensions, dwell time distributions, transition probabilities, and long-range temporal correlations of experimental EEG data can be captured (Creaser et al., 2021).

When, in addition to noise, deterministic external input is also included in the model, together with a function of finite-state computations (Ashwin and Postlethwaite, 2018), it is shown that the so-constructed network attractor can indeed perform finite-state computations. Mathematically, the model amounts to a nonlinear stochastic differential equation, where deterministic (external signal) and stochastic (noise) input are applied to any element. The deterministic input is determined by a symbol on a finite-state Turing machine. The accuracy and speed of computation depend on whether the system runs in the excitable or heteroclinic regime: The heteroclinic one is extremely sensitive to input and can be used as long as the input dominates noise. A reduction in the excitability is analogous to a neuromodulator that depresses excitability, reduces the speed of computation, and changes the error rates. This may shed some light on the effect of neuromodulators on cognitive functions such as computations via the brain. Thus, on one hand, the model allows us to analyze the competition between heteroclinic transitions and excitable connections between states, the heteroclinic transitions occurring spontaneously with characteristic dwell times in the vicinity of saddles, and the excitable ones requiring a finite threshold to be overcome for a transition to another state. On the other hand, the model allows us to study the competition between noise and external input and its impact on properties of computation such as speed and error rates. In spite of characteristic differences, common to both realizations, faster performance goes along with more errors, while fewer errors occur with slower performance, including some counterintuitive behavior of non-monotonic dependence as a function of noise.

Artificial recurrent neural networks are appreciated for their utility to recognize speech or handwriting or time series forecasting, and nowadays, techniques for training these networks make use of reservoir computing with echo state networks as a special case (Jaeger et al., 2007; Maass et al., 2007). Usually, these methods from machine learning amount to a black box in the sense that one does not understand the “strategy” of how the machine finds the solution. Neither understood are possible similarities to how the brain finds the solutions for problems, such as recognition, so fast and efficiently. Here, an attempt to understand the recurrent neural network behavior was made by Ceni et al. (2020) by proposing an algorithm to extract excitable network attractors from the trajectories generated by the neural networks upon solving tasks. The extracted network attractors live in a dimensionally reduced space and should reveal a coarse-grained description, thus providing an understanding of how a solution is achieved: exploiting long transients for prediction or fast switching for classification tasks and the like, basic “strategies” or mechanisms for solving tasks.

### 4.2 Designing deterministic chaotic heteroclinic networks

When activities in brain networks are manifest in transitions between metastable states that correspond to different types of behavior, or decisions, or other dominant biological states, these transitions look often quite random as if driven by a source of random noise. A first approach to reproduce random transitions is via Markov chains as a stochastic description. However, even if the switching statistics would be correctly reproduced in Markov models, they can neither explain the cause of the switching nor the variation in the target state with the dwell time (see, for example, the work of Nichols et al. (2017)). Moreover, the Markov assumption may not be applicable if the transitions depend on the history of the sequence. At a first place, one may think of a deterministic description complemented by additive noise, such as noisy HNs mentioned in Section 4.1; the role of noise is considered in more detail in Section 5.

Yet, there is a deterministic alternative. From dynamic systems theory, it is well known that what looks random may result from fully deterministic but underlying chaotic dynamics (chaos can act like a perfect random number generator; see, for example, the more recent work by Kaszás et al. (2019)). Such an approach of chaotic HNs was pursued by Morrison and Young (2022). Morrison and Young (2022) succeeded in reproducing the switching dynamics of *C. elegans* as experimentally measured in laboratory experiments,^3^ although the switching events between some simple types of behaviors look quite random. Examples for these behaviors are forward crawling, turns, reversals, and quiescence in the case of *C. elegans*, and the metastable states represent transient excited subpopulations of neurons which represent these specific behaviors. The transitions seem not to be evoked by external stimuli as they appear mostly spontaneously.

Morrison and Young (2022) provide a constructive description of how to tailor a chaotic HN that can reproduce the dwell times in the vicinity of the saddle equilibria, or the branching probabilities when decisions are taken, or other features of the transition statistics. The construction is based on maps (discrete in time) of two-dimensional flows of a vector field, where heteroclinic orbits connect saddle-fixed points in an HN along with the geometry of stable and unstable manifolds and their *non-trivial* intersections to control the switching dynamics. Starting from a desired set of saddle-fixed points, global dynamics are composed by interpolating local dynamics $f_{i}\left(\vec{x}\right)$ with weighting functions $w_{i}\left(\vec{x}\right)$ according to $d\vec{x}/dt=\sum_{i=1}^{m}w_{i}\left(\vec{x}\right)f_{i}\left(\vec{x}\right)$ with $\vec{x}=\left(x,y\right)\in R^{2}$ , and functions $w_{i}\left(\vec{x}\right)$ , which weight the local dynamics in each local dynamics region, and *i* labels these regions with *m* regions in total. A two-dimensional map is then constructed from this continuous time version, and deterministic perturbations are applied to the heteroclinic orbits prior to each stable direction of a fixed point in the map. Due to the designed perturbations, the unstable manifolds of saddle *ξ*_*i*_ and stable manifold of saddle *ξ*_*i*+1_ intersect non-trivially and allow for branching behavior of the trajectory toward the up-or-down branch of the unstable manifold of *ξ*_*i*+1_.

So far, the construction by Morrison and Young (2022) dealt with simple saddle equilibria and may be generalized to heteroclinic connections between hyperbolic invariant sets such as periodic orbits, invariant tori supporting quasi-periodic motion, or chaotic dynamics themselves. Alternatives for implementing the option of branching to those by Morrison and Young (2022) exist and are realized via a selected choice of eigenvalues at the saddles with high-dimensional unstable manifolds (see, for example, the work of Voit and Meyer-Ortmanns (2018), for two-dimensional unstable manifolds). Currently, it seems open whether a successful effective modeling via chaotic HNs reveals the actual physical mechanisms behind the irregularly looking switching dynamics, but what looks random may indeed result from deterministic rules, and there is a chance to uncover such rules via this kind of modeling.

## 5 Heteroclinic networks under the action of noise and external forcing

The role of noise in HNs may be rather subtle even if it is weak. In general, it has a strong impact on the period of heteroclinic oscillations, and the average dwell time in the vicinity of a saddle scales as a function of the noise amplitude. Often, for weak noise, its role is to prevent the slowing down of heteroclinic motion as the stochastic fluctuations keep the trajectory at some distance from the saddle equilibria. Furthermore, noise may facilitate synchronization of coupled heteroclinic units (Thakur and Meyer-Ortmanns, 2022) if it prevents a detailed resolution of the fine structure of the attractor landscape. This observation provides an explanation of the result obtained by Rana and Barato (2020) on the thermodynamic cost for achieving precise patterns. Rana and Barato (2020) addressed the relation between a stochastic Turing pattern in a Brusselator model and the thermodynamic cost: the precision of the patterns is maximized for an intermediate thermodynamic cost that is paid for the suppression of fluctuations. This means that tolerating a certain strength of fluctuations can have a positive effect on the precision of the pattern. This may be due to the fact that an intricate substructure of the attractor space is not resolved in the presence of noise, so the trajectories of different units become more similar in phase space and synchronize more easily to produce precise patterns.

The strength of noise can also act as a control parameter, similar to a bifurcation parameter. In the example of a hierarchical HN, an increase in the noise strength changed the dynamics from two to one hierarchy level to a global coexistence equilibrium. Similarly, it influences the entrainment range of pacemakers: the stronger the noise, the more difficult the entrainment to heteroclinic oscillations becomes (Thakur and Meyer-Ortmanns, 2022).

So far, we referred to actual observations of the role of noise in concrete systems. In general, care is needed even for very small noise strength, as careful analyses (Stone and Armbruster, 1999; Armbruster et al., 2003; Bakhtin, 2010; Ashwin and Postlethwaite, 2013; Manicom, 2021) have previously shown. Stone and Armbruster (1999) considered HCs connecting saddle equilibria with one-dimensional unstable manifolds under the influence of noise. They derived a Fokker–Planck equation for the evolution of the probability distribution of trajectories near HCs. Solving the Fokker–Planck equation showed the impact of the stable and unstable eigenvalues at the fixed points and the impact of noise on the location and shape of the probability distribution. The probability distribution then explains the noise-induced jumping of solution trajectories in and out of invariant subspaces of the deterministic system.

More generally, Armbruster et al. (2003) analyzed the influence of small noise on the dynamics of HNs with a focus on noise-induced switching between cycles within the network. Three different types of switching are distinguished: random switching between heteroclinic cycles, determined by the linearized dynamics near one of the saddles; counterintuitive noise-induced stability of a cycle; and intermittent switching between cycles. Essential are the size of stable and unstable eigenvalues at the saddle equilibria. What may happen close to equilibria in the presence of noise is a so-called lift-off from the coordinate axes in the probability distribution of outgoing trajectories, moving past a saddle-type equilibrium. This means that the bundle of trajectories making up the distribution is lifted away from the noise-free heteroclinic connection as a result of a positive saddle quantity $\sigma ≔\lambda_{u}+\lambda_{s_{n}}$ with eigenvalues $\lambda_{s_{1}}<\lambda_{s_{2}}<\ldots <\lambda_{s_{n}}<0$ and *λ*_*u*_ > 0 at the saddle. Lift-offs change the intersections of the distribution of incoming trajectories into the neighborhood of a saddle and outgoing trajectories to a given next saddle. This way, they can induce memory in the sense that the transition probability between vertices depends not only on the current vertex but also on the past few vertices visited previously. On the other hand, if the transition probabilities are independent of the prior itinerary, an attracting HN is memoryless under the action of noise; in this case, the dynamics for low noise amounts to a one-step Markov process.

Since the presence or absence of this specific type of memory can be controlled by tuning the size of the contracting versus expanding eigenvalues, a possible application in view of cognitive processes is task switching. Starting or switching the attention between tasks is common to executive functions in cognitive processes that define behavior such as learning, paying attention, organizing, or planning. Task switching depends on memory as the performance of one task depends on an earlier task. Manicom (2021) used a model of task switching that makes use of a mixed heteroclinic and excitable network with non-autonomous input, which is shown to produce a similar memory effect to noise. In particular, the time it takes to complete a cycle of the network depends on whichever cycle was most recently completed.

In summary, depending on how noise interacts with the underlying deterministic dynamics of an HN, it may lead to a one-step Markov process, or to long-term memory in the mathematical sense (of dealing with a non-Markovian process). Results on this subtle role are important in view of designing HNs for controlling decisions at branching points, i.e., at saddles with high-dimensional unstable manifolds. For the control, eigenvalues should be accordingly tuned together with noise amplitudes or entire noise distributions.

Apart from the response to noise, external forcing plays an important role for HD as the very selection of specific heteroclinic sequences is supposed to result from external input. Usually, for attractive coupling of nonlinear oscillators, a weak periodic external force can lock a nonlinear oscillator at a frequency close to the input frequency while for stronger forcing also at subharmonic bands. In contrast, once competition is included, such as for GLV equations, with a weak external signal, synchronization of ultra-subharmonics is dominant over the frequency close to that of the input. A forced system near a heteroclinic orbit seems to be very flexible to lock in a wide range of ultra-subharmonic frequencies (Rabinovich et al., 2006d). These results suggest speculating that the observed synchronization behavior is at the origin for synchronization between experimentally observed slow and fast brain rhythms (Tass et al., 1998; Palva et al., 2005). A more detailed analysis of the same system by Tsai and Dawes (2011) shows that it is the ratio of contracting to expanding eigenvalue *δ* = *c*/*e* that determines whether this flexible frequency locking is observed or not. For *δ* ≫ 1, no frequency locking is observed; for intermediate *δ*, the results obtained by Rabinovich et al. (2006d) are confirmed; and for *δ* ≃ 1, the dynamics resembles a forced damped pendulum.

## 6 Learning in heteroclinic dynamics

We discussed synchronization of heteroclinic networks in Sections 3.3, 3.4. As mentioned previously, synchronization is usually achieved via the transmission of signals from one oscillator to another by coupling the state variables. In contrast, Selskii and Makarov (2016) showed that synchronization of HNs results from learning in coupled neural networks. A driver network (the master or teacher) exhibits winnerless competition dynamics, while a driven network (the slave) tunes its internal couplings according to the oscillations observed in the teacher. The observation follows a learning rule that includes memory effects, as the incoming information is integrated over some time. This way, the learner can learn the coupling structure from the teacher and synchronize with the teacher by adapting the expanding eigenvalues only. The learning works for an intermediate memory length. Tapia et al. (2018) extended this approach by an additional step that identifies the sequence of saddles in a discrete manner but is still limited to circular topologies.

Most of the models of associative sequential memory are based on the generalization of the Hopfield associative memory network (Hopfield, 1982). However, Seliger et al. (2002) showed that the learning dynamics leads to the formation of a winnerless competition network that is capable of the associative retrieval of pattern sequences which were recorded previously. The model of the sequential spatial memory in the hippocampus is implemented in a two-layer neuronal structure, where the first layer serves as a sensory input for the second layer, which performs winnerless competition among representative principal neurons. The learning mechanism that alters and adapts the competition matrix is realized via delay differential equations.

Learning from a teacher in view of inference of an underlying dynamics (that may have produced a given dataset) was considered by Voit and Meyer-Ortmanns (2019b), who showed how the topology and connection strength of an HN can be inferred from data in a dynamical way. A template system is unidirectionally linearly coupled to the input in a master–slave fashion so that it is forced to follow the same sequence of saddles which are approached by the master. At the same time, its eigenvalues are adapted to minimize the difference of template dynamics and input sequence. The dynamics of a master and slave may be different, but the template system learns to mimic an input sequence of metastable states under the assumption that the data were generated by HD.

## 7 Conclusion and perspectives

As mentioned, the framework of HD is abstract enough that the basic variables may represent the firing rates of single neurons or groups of neurons on different time scales and from different brain areas. The combination of excitatory and inhibitory interactions within and between different groups of neurons admits solutions which can, in principle, reproduce features of typical switching patterns of metastable states, as observed in experiments. The sensitivity to external input and the subtle role of noise allow an adaptation of the switching statistics to experimental data, characterized by dwell times and transition probabilities between metastable states. As a very promising extension of differential equations with a heteroclinic attractor appears a set of stochastic differential equations that also includes an excitable attractor in phase space. Sets of stochastic differential equations can be designed to reproduce a network whose nodes correspond to experimentally identified metastable states and whose edges represent heteroclinic or excitable connections, which are assumed to mediate the transitions. When details of the switching statistics, such as heavy-tailed dwell time probability distributions, should be captured, a more nested set of differential equations may be needed rather than a simple set of GLV equations.

So far, we discussed disorder as induced by additive or multiplicative noise. Other sources of disorder and their role in HNs may be further explored, such as heterogeneous choices of parameters such as rates of birth, decay, or competition strengths. Further types of spatial couplings of HNs beyond diffusive coupling should be considered in view of controlling partial synchronization and information processing across spatial grids.

Moreover, a new aspect from the physics perspective is worthwhile being pursued. Heteroclinic dynamics is appreciated for combining features of precise reproducibility with a high sensitivity to external input. What is the behavior if the external input suddenly changes and a fast response to the new input is required? This question was recently addressed by Aravind and Meyer-Ortmanns (2023). Aravind and Meyer-Ortmanns (2023) assumed external input to be encoded in the very selection of the rates in the competition rate matrix. New external input is realized as a quench of the bifurcation parameter from a regime of heteroclinic oscillations to a regime of equilibrium states. This way, the question is reduced to measure the relaxation time that the system needs to arrest its oscillations toward a resting state. The relaxation turns out to be underdamped and to depend on the size of the attractor basin, the depth of the quench, the level of noise, the nesting of the attractor space, and the coupling type, strength, and synchronization. This means that the relaxation time can be pronounced. Here, we speculate that a possible manifestation of underdamped heteroclinic motion may be visible in a malfunction of brain dynamics, for example, when heteroclinic cycles dynamically realize gaits and an undesired delay is observed in arresting motion at wish. Such a delay is one of the symptoms of Parkinson’s disease. Therefore, investigations of how to control relaxation times and fast adaptation to new input in HD deserve further attention (Aravind and Meyer-Ortmanns, 2023).

In view of cognitive processes, it is most interesting to extend analyses of how learning can be realized in HD, in particular and as a further step the thermodynamic cost for learning. Based on results obtained by Goldt and Seifert (2017a) and Goldt and Seifert (2017b), one would expect that the information that can be acquired by learning via HD will be bound by the thermodynamic cost of learning, similar to the cost for Hebbian learning, which restrains the accessible information. Beyond the application to learning, future modeling of HD (possibly in combination with excitable dynamics in phase space) should include the cost in terms of energy usage, “disc space”, and time, as limited disc space may require overwriting and time enters the performance speed. A reliable performance in the presence of noise and at a reasonable speed is certainly not for free. The tradeoff between precision in performance when high precision is needed, speed of its execution, and cost for both performance and maintenance of function should be well balanced. It is an open and fascinating question as to how the brain achieves such a balance over a long time span in spite of error-prone reading, writing, and overwriting on the finite “disc” of the brain.

## Footnotes

1 What is specific for a coupling of HUs on a *spatial* grid is coupling via diffusion between repetitive units; an alternative interpretation of this set is a system of specifically coupled HUs in a large phase space (assigned to a single site).

2 Note that the excitable network here refers to a network in phase space, where connections between states in phase space become possible when a finite threshold is overcome. Excitable networks in ordinary space, such as a two-dimensional grid, refer to individually excitable cells, coupled across the grid; an example of such a network is considered by Labavić and Meyer-Ortmanns (2014) for repressive coupling.

3 This formulation is a shortcut to a sequence of preceding steps. Experimental measurements of neural activity in *C. elegans* are based on whole-brain calcium imaging with cellular resolution (Kato et al., 2015; Nguyen et al., 2016), simultaneous whole-animal 3D imaging of neural activity by light-field microscopy (Prevedel et al., 2014), or brain-wide 3D imaging of neural activity with sculpted light (Schröd et al., 2013). As a next step, such measured activity was represented in low-dimensional PCA space, where metastable states correspond to stereotypical behaviors of *C. elegans* with probabilities of transitions between these states (Kato et al., 2015; Nichols et al., 2017; Linderman et al., 2019). Only after these preceding steps, the work of Morrison and Young (2022) aims at reproducing characteristic features of the PCA results: for transition probabilities between states from the work of Nichols et al. (2017) and indications on dwell times from the work of Linderman et al. (2019).
