# Supplementary material for: Heteroclinic networks for brain dynamics
Source: Front Netw Physiol. 2023 Nov 8;3:1276401. doi: 10.3389/fnetp.2023.1276401 (PMC10663269; doi:10.3389/fnetp.2023.1276401)
Supplement: Supplementary file 1 [file Presentation1.pdf]

Heteroclinic networks for brain dynamics: Supplementary material

Hildegard Meyer-Ortmanns [1,2*]

[1] *School of Science, Constructor University, Bremen, Germany*
[2] *Complexity Science Hub Vienna, Vienna, Austria*

Correspondence*:
Corresponding Author
hortmanns@constructor.university

1 APPENDIX TO THE MAIN TEXT

1.1 Definitions of Heteroclinic Networks

Various definitions of HNs are used in the literature. The definition of a HN as used in (1) is the following: Consider an ODE with phase space $x \in \mathbb{R}^d$, defined by

$$dx/dt = \dot{x} = f(x), \tag{1}$$

and suppose that the flow generated by the solution of this ODE starting at x_0 is $x(t) = \phi_t(x_0)$. Let $B_\delta(x)$ denote the closed ball centered on x with radius $\delta > 0$. For ξ an equilibrium of 1 define the stable and unstable sets $W^s(\xi) = \{y : |\phi_t(y) - \xi| \to 0$ as $t \to \infty$ and $W^u(\xi) = \{y : |\phi_t(y) - \xi| \to 0$ as $t \to -\infty$; these are manifolds if ξ is hyperbolic. A set $X \subset \mathbb{R}^d$ is a (weak) *heteroclinic network* (in phase space) if there is a set of equilibria $\{\xi_i\}_{i=1}^n$ such that

$$X = X_{het}(\{\xi_i\}) := \bigcup_{i,j=1}^{n} W^u(\xi_i) \cap W^s(\xi_j) \tag{2}$$

and there is a heteroclinic connection from ξ_i to ξ_j whenever

$$W^u(\xi_i) \cap W^s(\xi_j) \neq \emptyset. \tag{3}$$

No homoclinic connections are assumed. According to (1), this definition of a HN is substantially weaker than that given in most of the literature such as those of (2), (3), as, for example, no chain recurrence or connectedness of the network are required. For further definitions and their difference we refer to (1), (4), or (5) as well as to (6),the latter one giving a so-called intrinsic definition of a HN.

1.2 Notions of stability

We also want to point to subtleties in the stability properties. The overall goal is to control stability properties along heteroclinic connections which turn out to be involved, in particular for non-hyperbolic equilibria. In applications to neural (or genetic) networks such stability properties matter when asking in what sense a subnetwork is apparently stable though it is part of a larger HN and itself cannot be asymptotically stable. An example for a non-asymptotically stable attractor was given by I. Melbourne (7), where nodes on the HC have unstable eigenvalues in directions ‘normal’ to the cycle, and yet the cycle is stable in the sense that the only trajectories that do not remain close to the invariant set but escape, lie in a cuspoidal region, abutting the invariant set. This kind of stability is termed essential asymptotic stability (e.a.s.). A further notion is fragmentary asymptotic stability (f.a.s.). For the definition of both notions see, for example, (8) or (9). These notions are particularly useful for non-hyperbolic equilibria and further characterize the very set that attracts trajectories from initial conditions of positive measure (f.a.s.) or almost all initial conditions (e.a.s.) from a small neighborhood of the invariant object such as a HC. For example, the property of e.a.s. may explain numerical observations that HCs coexist, which depending on the initial conditions lead to a pair of standing waves or rotating waves. Both cannot be asymptotically

stable at the same time, if the initial conditions are chosen close to each other, but they are essentially asymptotic stable. In more general terms this means that even small neighborhoods of invariant objects such as HNs may have nontrivial substructures with respect to their attraction properties. Their existence is not naively expected, but may have observable implications in “real-life” situations, as stated in (7).

REFERENCES

[1]Ashwin, P. and Postlethwaite, C., 2016. Designing heteroclinic and excitable networks in phase space using two populations of coupled cells. Journal of Nonlinear Science, 26, pp.345-364.

[2]Kirk, V., Postlethwaite, C., and Rucklidge, A. M., 2012. Resonance bifurcations of robust heteroclinic networks. SIAM Journal on Applied Dynamical Systems, 11(4), pp.1360-1401.

[3]Ashwin, P. and Postlethwaite, C., 2013. On designing heteroclinic networks from graphs. Physica D: Nonlinear Phenomena, 265, pp.26-39.

[4]Field, M. J., 2015. Heteroclinic networks in homogeneous and heterogeneous identical cell systems. Journal of Nonlinear Science, 25(3), pp.779-813.

[5]Field, M. J., 2016. Patterns of desynchronization and resynchronization in heteroclinic networks. Nonlinearity, 30(2), pp.516-570.

[6]Ashwin, P. and Field, M., 1999. Heteroclinic networks in coupled cell systems. Archive for Rational Mechanics and Analysis, 148, pp.107-143.

[7]Melbourne, I., 1991. An example of a nonasymptotically stable attractor. Nonlinearity, 4(3), pp.835-844.

[8]Podvigina, O., 2012. Stability and bifurcations of heteroclinic cycles of type Z. Nonlinearity, 25(6), pp.1887-1917.

[9]Lohse, A., 2022. Stability indices of non-hyperbolic equilibria in two-dimensional systems of ODEs. Dynamical Systems, 37(4), pp.699-709.
